# Effects of linear versus curvilinear sprint training on multidirectional speed in young soccer players: a randomized parallel-group trial

**DOI:** 10.5114/biolsport.2025.139084

**Published:** 2024-05-24

**Authors:** David Solleiro-Duran, Pablo Cidre-Fuentes, Ezequiel Rey, Andrés Baena-Raya, Alberto Filter, Alexis Padrón-Cabo

**Affiliations:** 1 University of A Coruña, Department of Physical and Sports Education, Faculty of Sport Sciences and Physical Education, A Coruña, Spain; 2 High Performance Department, Olympique de Marseille, Marseille, France; 3 University of Vigo, Faculty of Education and Sport Sciences, Pontevedra, Spain; 4 Department of Education, Faculty of Education Sciences, University of Almería, Almería, Spain; 5 SPORT Research Group (CTS-1024), CIBIS (Centro de Investigación para el Bienestar y la Inclusión Social) Research Center, University of Almería, Almería, Spain; 6 FSI Lab, Football Science Institute, Granada, Spain; 7 Research Group Physical Activity, Health and Sport CTS-948, Pablo de Olavide University, Sevilla, Spain

**Keywords:** Football, Curved sprint, Force-velocity profile, Acceleration, Physical fitness

## Abstract

The purpose of this study was to examine the effects of linear sprint training (LST) compared to curvilinear sprint training (CST) using an equivalent session training volume, on linear (LS) and curvilinear (CS) sprint performance, horizontal force-velocity profile, and change of direction (COD) ability in young soccer players. In a randomized pre-post parallel-group trial design, nineteen U16 male soccer players were randomly assigned to LST (n = 9) and CST (n = 10) groups. Both groups performed 11 sprint training sessions over 6 weeks. Before and after the training period, LS (5 m, 10 m, 15 m, 20 m, and 30 m), CS (weak and good side), horizontal force-velocity profile, and COD speed (modified 505 test) were assessed. The LST showed small to moderate significant enhancements (p ≤ 0.05) in LS (except 5 m sprint), modified 505 test, theoretical maximal velocity (V_0_), maximal power output (P_max_), and maximal ratio of force (RF_peak_) from pre-test to post-test. CST resulted in small to moderate significant improvements (p ≤ 0.05) in 10 m, 20 m, and 30 m LS performance, weak and good sides of the CS, COD speed, and P_max_ from pre-test to post-test. In addition, significant between-group comparisons were observed between LST and CST for CS performance in both sides (p < 0.01). In conclusion, LST and CST seem to produce trajectory-specific adaptations in young soccer players. Therefore, strength and conditioning coaches should integrate both LST and CST training methods to effectively prepare soccer players and enhance their sprinting performance.

## INTRODUCTION

Soccer is a team sport characterized by intermittent bouts of sub-maximal running distances interspersed with high-intensity efforts, including maximum sprints [1]. Recent trends in professional soccer reveal a significant increase in high-intensity running distances [2, 3], with sprint distances in the English Premier League rising by 30–50% between 2006/2007 and 2012/2013 [4, 5, 6]. Additionally, sprint actions play a crucial role in soccer, frequently preceding goal situations [7]. Indeed, sprint ability has proven to be a differentiating factor among soccer players of different competition standards [8] and age categories [9, 10].

During soccer matches, sprint actions are not limited to linear sprint (LS) trajectories. Recently research by Caldbeck & Dos’Santos [11] found that approximately 85% of maximum velocity manoeuvres in professional adult men’s soccer involve curvilinear sprints (CS). Briefly, CS can be defined as “the upright running portion of the sprint completed with the presence of some degree of curvature” [12], typically within a radius ranging from 3.5 to 11 m in soccer [13]. Both LS and CS share a variation between 34% to 37% among experienced adult men soccer players, indicating distinct physical capabilities [14]. Specifically, CS requires the ability to generate centripetal forces, involving not only different kinetic [15] and kinematic [16] characteristics than LS, but also different neuromuscular activity in the outside and inside leg in semiprofessional adult men soccer players [16]. The inside leg’s adductor and semitendinosus muscles exhibit higher electromyography (EMG) activity, while the gluteus medius and biceps femoris muscles of the outside leg demonstrate increased activation [16]. Consequently, both actions seem to be trained separately to target the specific adaptations.

An emerging area of research in soccer is the categorization of sprint training modes based on task specificity. These modes provide a valuable framework for optimizing athletic performance, being classified into primary (e.g., sprint technique, unresisted sprint), secondary (e.g., resisted or assisted sprinting), and tertiary (e.g., non-specific methods such as resistance training and plyometrics) categories [17, 18]. From a performance perspective, Nicholson et al. [17] questioned the effectiveness of primary methods in improving short-sprint performance in soccer players and suggested that this effectiveness may also be influenced by mediator variables such as age or maturation status. Importantly, the role of the sprint trajectory (linear vs. curvilinear) within unresisted sprint training remains unknown. Similarly, another research gap in the current literature relies on understanding whether CS training impacts the mechanical capabilities of the neuromuscular system to produce force during LS. This ability is well summarized through the horizontal force-velocity (F-V) profile, which integrates the theoretical maximal force (F_0_), velocity (V_0_), maximal power output (P_max_), maximal ratio of force applied in the forward direction at the sprint start (RF_peak_), and the athlete’s ability to maintain a net horizontal force production despite increasing running velocity (D_RF_) [19].

Because sprinting has been considered a crucial ability in soccer [7, 11], understanding the manipulation of load factors (e.g., intensity, trajectory, frequency) in primary sprint training methods seems to be necessary. As mentioned previously, the effects of sprint trajectory manipulation (linear vs. curvilinear) in youth soccer players remain unknown. Consequently, there is a notable research gap regarding the effects of curvilinear sprint training on key parameters of the horizontal F-V profile and sprint performance in youth soccer players. Addressing these gaps could help strength and conditioning coaches to individualize and optimize sprint training interventions. Therefore, the aim of this study was to analyse the effects of linear sprint training (LST) compared to curvilinear sprint training (CST) using an equivalent session training volume, on LS and CS performance, horizontal F-V profile, and change of direction (COD) ability in young soccer players. In line with the principle of specificity, we hypothesized that CST would provide greater effects in CS performance and COD ability, and LST would induce greater improvements in different LS distances and the horizontal F-V profile.

## MATERIALS AND METHODS

### Experimental Approach to the Problem

A randomized pre-post parallel group trial design was used to assess the effect of 6 weeks of LST and CST intervention (twice a week) on sprint performance, COD, and F-V profile in U-16 youth soccer players. To ensure that participant allocation was unbiased, a computer-generated sequence was used for randomization. After the randomization procedure, the participants were assigned to either the linear (LSG) or the curvilinear (CSG) sprint group. During the mid-season period (the year 2023), the sprint intervention programmes were integrated into the regular soccer training routine of each group (Table 1). Two familiarization sessions were carried out before the baseline testing session to acquaint participants with the testing protocol. The following multidirectional speed tests were performed: (a) 5 m sprint, (b) 10 m sprint, (c) 15 m sprint, (d) 20 m sprint, (e) 30 m sprint, (f) CS, and (g) modified 505 test. To reduce the potential influence of confounding variables, all participants were instructed to uphold their usual lifestyle and dietary intake before and during the intervention. In addition, to minimize fatigue effects, all tests were performed after 72 hours of recovery from an official match or strenuous physical training. Baseline and post-testing sessions took place at the same time of the day (6–8 PM) to reduce the effects of circadian rhythms and under similar environmental conditions. Participants were instructed to wear the same athletic equipment during testing sessions.

**TABLE 1:** Training contents for each microcycle session.

Day	Description
MD+1	Rondos, medium-to-large sided possession games, small-to-medium-sided matches
MD-4	Strength training, small possession and position games, small-to-medium-side games
MD-2	Rondos, speed-agility training, pressing task, tactical drills and shooting drills
MD	Match day

MD+1: 1 day after match; MD-4: 4 days before match; MD-2: 2 day before match.

### Participants

Nineteen U16 male soccer players from the same soccer academy were recruited to this study. Previously to the recruitment procedure, a priori power analysis (G*Power, version 3.1.9.7, Universität Kiel, Düsseldorf, Germany) with an assumed type I error of 0.05 and a type II error rate of 0.20 (80% statistical power) was performed for sprint performance [20]. Based on the a priori power analyses, a minimum sample size of eight subjects per group would be sufficient to detect medium group × time interaction effects. Players were randomly assigned to the LSG (n = 9; age: 15.6 ± 0.5 years, height: 172.1 ± 6.9 cm; body mass: 65.7 ± 6.7 kg) or the CSG (n = 10; age: 15.9 ± 0.3 years, height: 174.9 ± 6.3 cm; body mass: 63.2 ± 6.8 kg). To be included in the present study, participants had to fulfil the following inclusion criteria: (a) a minimum of four years’ experience in systematic training and competition in soccer, and (b) no history of injuries within the three months prior to the intervention. During the intervention period, players regularly performed three training sessions and one official match per week. In addition, the final statistical analyses considered only those players who completed familiarization sessions, testing sessions, and at least 80% of all training sessions. After the explanation of the experimental protocol and its potential benefits and risks, the legal representatives provided written informed consent to participate in the current study. The research protocol and associated procedures were approved by the Local Ethics Committee (code: 04-280723). All procedures were conducted according to the Declaration of Helsinki for human studies [21].

### Testing Procedures

Prior to the pre-testing, two familiarization sessions were conducted in order to mitigate the potential learning effects. In these familiarization sessions, the participants received instructions about the correct execution of each experimental test. Afterward, the physical tests were performed in a single session 1 week before starting the training intervention on the same artificial grass soccer pitch. At the beginning of the pre-test session, prior to the standardized warm-up, the participants’ anthropometric measurements were taken. Specifically, the standing height was obtained using a fixed stadiometer (Holtain Limited, Crosswell, United Kingdom), and body mass was measured to the nearest 0.1 kg using a digital scale (BC-554 Ironman Body Composition Monitor, Tanita, Illinois, USA). Afterward, a standardized warm-up routine was established, including 5 min of low-intensity running, 5 min of dynamic stretching, and short progressive accelerations (four submaximal sprints, progressing to 90% of the players’ self-perceived maximal speed over 30 m distance). Additionally, the participants were encouraged by supervisors to execute maximal effort during physical tests.

### Linear Sprint Tests and Horizontal Force-Velocity Profile

Sprint times were measured using a system of six pairs of dual-beam photoelectric cells (Witty, Microgate, Bolzano, Italy), positioned at distances of 0, 5, 10, 15, 20, and 30 m. Players were instructed to initiate the LS from a standing start, with their preferred front foot placed 0.5 m behind the first timing gate. A verbal instruction of “sprint as fast as possible” was received by supervisors. To ensure reliable and consistent data, players were allowed two trials, with a 3 min recovery between them. Specifically, the fastest trial and their split times were recorded to analyse the LS test performance and variables derived from the horizontal force-velocity profile. Based on previous correction factors proposed in scientific literature [22, 23], 0.5 s was added to all sprint times for converting the first movement triggering. A custom-made spreadsheet developed by Morin & Samozino [24] was used to calculate force-velocity derived variables. This spreadsheet employs a monoexponential time-velocity function to fit raw time-position data. The main mechanical variables obtained from the horizontal force-velocity profile were [19]: F_0_, V_0_, P_max_, the slope of the linear F-V relationship (FV slope), RF_peak_, and D_RF_. Moreover, data about temperature and atmospheric pressure were collected.

### Curve Sprint Test

According to Fílter et al. [12], the CS test was established as reliable. In line with the aforementioned research, the curve trajectory corresponds to the arc of the penalty area of an official soccer field. This curve trajectory was standardized as follows: (a) a 9.15 m radius from the penalty spot, (b) covering a distance of 14.6 m from starting point to the final point of the curve in a straight line, and (c) forming an angle of 105.84° of amplitude from the penalty spot. Derived from trigonometrical analyses, sprint time was measured for players over 17 m during the CS test. To measure CS time, photoelectric timing gates (Witty, Microgate, Bolzano, Italy) were positioned at the starting and final points of the curved trajectory. All players began from a standing start, with the front foot positioned 1 m from the first timing gate [12]. Two trials were completed for each side, separated by 5 min resting period [25]. The sides were categorized as “good” and “weak”. Specifically, the “good” side corresponds with the fastest time recorded, while the “weak” side indicates the slowest time recorded. These categorizations were determined based on the best time trial obtained for each respective side.

### Modified 505 Test

A photoelectric cells system (Witty, Microgate, Bolzano, Italy) was used to record the performance in the modified 505 test [26]. In this test, players began from a standing start with their preferred foot 0.5 m behind the timing gate. When participants received an audio signal start, they sprinted through the timing gate, reached the 5 m turn line, executed a 180º COD with their dominant leg, and sprinted back 5 m through the timing gate. To ensure data consistency, a supervisor was placed at the turning line. If a player performed the COD before the turning point, the trial was discarded and retried after a recovery period. All participants completed three trials with 3 min recovery time between them. Furthermore, the COD deficit (COD_def_) for the modified 505 test was calculated as follows: 505 COD time – 10 m sprint time [27]. The COD deficit calculation was applied to provide a more isolated measure of COD ability, reducing the influence of acceleration and LS ability [27].

### Training Programme

The intervention programme took place during the mid-season period. After pre-testing, the 6-week sprint protocol (a total of 11 sessions) was performed twice during the first 5 weeks with at least 48 hours of recovery period between sessions. In the last week of the training intervention, a tapering procedure was planned to gradually reduce the total distance covered by both groups. Both groups were exposed to the same distance per repetition, session, and week (Table 2). Additionally, all players were instructed to provide maximal effort (i.e., all-out mode) during each LS and CS repetition [28, 29]. According to previous research, young male athletes are capable of reliably regulating their performance during high-speed forward running (> 90% maximal effort) [30]. The main difference between the 2 intervention groups was the trajectory: the LSG executed all sprints in a straight line, while the CSG performed sprints with a curved trajectory. In this regard, Altmann et al. [31] found very large to nearly perfect associations (r = 0.79–0.91) of linear sprint time performance with three different CS radii (i.e., narrow angle: 7.15 m; medium angle: 9.15 m; wide angle: 11.15 m). Consequently, to standardize the curved trajectory for the CSG group, all CS were performed around the penalty arc (i.e., 105.84° amplitude) on a soccer pitch with official dimensions. To maintain a homogeneous stimulus between both legs (i.e., good and weak sides), players executed the same number of repetitions starting from the different sides (i.e., clockwise and anticlockwise paths) of the penalty arc. All sessions took place on the same artificial pitch turf. Before each intervention session, the participants followed the same standardized warm-up routine performed during the testing sessions. A certified strength and conditioning coach supervised each warm-up routine and sprint training session, providing verbal feedback and encouraging players to give a maximal effort.

**TABLE 2:** Descriptive characteristics of the sprint training program performed by both experimental groups (linear sprint *vs.* curve sprint).

Weeks	Sessions per week	Distance per repetition (m)	Repetitions	Sets	Total distance per training (m)	Total distance per week (m)	Recovery between repetitions (min)	Recovery between sets (min)
1	2	17	6	1	102	204	2	5
2	2	17	4	2	136	272	2	5
3	2	17	5	2	170	340	2	5
4	2	17	3	4	204	408	2	5
5	2	17	3	4	204	408	2	5
6	1	17	3	4	204	204	2	5

### Statistical Analyses

Data are presented as mean ± standard deviation (SD). The normality assumption was checked both graphically and through the Shapiro-Wilk test, and it was found that all analysed variables displayed a normal distribution. A 2 (Time: Pre vs. Post) × 2 (Group: LSG vs. CSG) repeated measures analysis of variance (ANOVA) with Bonferroni post hoc corrections was conducted to analyse the effects of sprint training protocols for each performance variable. In this analysis, the partial eta squared (*η*_p2_) was calculated and interpreted as follows: ≥ 0.01 indicates a small effect, ≥ 0.059 a medium effect, and ≥ 0.138 a large effect. Additionally, effect sizes were determined using Cohen’s *d* with the following formula: *d* = (M_2_ – M_1_)/SD_pooled_. According to Hopkins et al. [32], effect sizes were categorized as trivial (d *<* 0.2), small (0.2 ≤ *d <* 0.6), moderate (0.6 ≤ *d < 1*.2), large (1.2 ≤ *d <* 2.0), and very large (≥ 2.0). Also, the within-sessions reliability was analysed using the intraclass correlation coefficient (ICC_3,1_) and coefficient of variation (CV) and interpreted according to Hopkins et al. [32]. All statistical analyses were conducted using the statistical software SPSS for Macintosh (version 25.0; Armonk, NY: IBM Corp). The significance level for all analyses was set at *p* ≤ 0.05.

## RESULTS

Training compliance was 94% for the LSG and 90% for the LSG and CSG. Within-session reliability is shown in Table 3. For all fitness tests, relative reliability (ICC_3,1_) ranged from high to extremely high values for both groups. Furthermore, a CV of less than 10% was achieved, indicating acceptable absolute reliability.

**TABLE 3:** Within-session reliability for each physical fitness test before and after sprint training.

	Pre		Post	

Fitness Test	ICC_3,1_(95% CI)	CV	ICC_3,1_(95% CI)	CV
5 m sprint (s)	0.86 (0.71–0.93)	2.49	0.76 (0.54–0.89)	2.61
10 m sprint (s)	0.94 (0.87–0.97)	1.43	0.98 (0.97–0.99)	0.53
15 m sprint (s)	0.96 (0.92–0.98)	1.06	0.98 (0.95–0.99)	0.69
20 m sprint (s)	0.97 (0.93–0.99)	1.01	0.98 (0.97–0.99)	0.56
30 m sprint (s)	0.98 (0.95–0.99)	0.85	0.98 (0.97–0.99)	0.61
Curve Sprint Good Side (s)	0.94 (0.86–0.97)	1.08	0.91 (0.80–0.96)	1.71
Curve Sprint Weak Side (s)	0.94 (0.87–0.97)	1.03	0.96 (0.92–0.98)	0.95
M505 Test (s)	0.70 (0.42–0.85)	2.31	0.80 (0.60–0.91)	2.04

Abbreviations: LSG: linear sprint group; CSG: curve sprint group; M505 Test: modified 5-0-5 change of direction test; ICC_3,1_: intraclass correlation coefficient; CV: coefficient of variation.

**FIG. 1:**
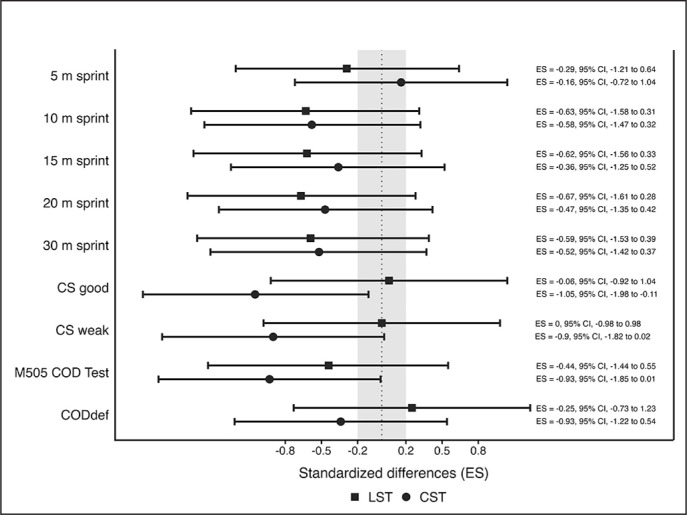
Standardized differences (95% CI) in all physical fitness variables between pre-and post-test for linear (filled squared) and curvilinear (filled circles) sprint training groups. LST: linear sprint training group; CST: curvilinear sprint training group; ES: effect size; CS: curvilinear sprint; M505 COD Test; modified 505 change of direction test; COD_def_: change of direction deficit.

Table 4 displays mean values, SD, and percentage change from pre- to post-intervention for the 5 m, 10 m, 15 m, 20 m, and 30 m sprint test, CS test, and modified 505 test. Additionally, Figure 1 shows the standardized differences between the pre-test and post-test for LST and CST groups.

**TABLE 4:** Changes in physical fitness after six weeks of sprint training in youth soccer players.

	LSG			CSG			ANOVA *P* values ($\eta_{\text{p}}^{2}$) ($\eta_{\text{p}}^{2}$ category)		

	Pre	Post	∆ (%)	Pre	Post	∆ (%)	Time	Group	Time × Group
**5 m sprint** (s)	1.61 ± 0.07	1.59 ± 0.07	-1.04	1.58 ± 0.08	1.59 ± 0.04	0.98	0.599 (0.017) *small*	0.550 (0.021) *small*	0.362 (0.049) *small*

**10 m sprint** (s)	2.41 ± 0.12	2.34 ± 0.10^**^	-4.13	2.37 ± 0.10	2.32 ± 0.07^*^	-2.17	< 0.001 (0.506) *large*	0.495 (0.028) *small*	0.291 (0.065) *medium*

**15 m sprint** (s)	3.10 ± 0.16	3.01 ± 0.13^**^	-3.93	3.04 ± 0.12	3.00 ± 0.10	-1.55	< 0.001 (0.553) *large*	0.568 (0.020) *small*	0.081 (0.169) *large*

**20 m sprint** (s)	3.76 ± 0.21	3.63 ± 0.18^**^	-3.83	3.72 ± 0.16	3.65 ± 0.14^*^	-2.10	< 0.001 (0.553) *large*	0.852 (0.002) *trivial*	0.223 (0.086) *medium*

**30 m sprint** (s)	5.03 ± 0.31	4.86 ± 0.27^**^	-4.13	5.00 ± 0.21	4.89 ± 0.21^*^	-2.23	< 0.001 (0.619) *large*	0.988 (0.001) *trivial*	0.169 (0.109) *medium*

**Curve Sprint Good Side (s)**	2.82 ± 0.13	2.83 ± 0.19	0.02	2.84 ± 0.10	2.73 ± 0.11^**^	-4.16	0.007 (0.371) *large*	0.526 (0.026) *small*	0.006 (0.390) *large*

**Curve Sprint Weak Side (s)**	2.90 ± 0.15	2.90 ± 0.17	0.13	2.87 ± 0.10	2.78 ± 0.10^**^	-3.36	0.009 (0.352) *large*	0.257 (0.080) *medium*	0.006 (0.390) *large*

**M505 COD Test (s)**	2.71 ± 0.13	2.65 ± 0.14^*^	-2.30	2.65 ± 0.10	2.57 ± 0.07^**^	-3.07	< 0.001 (0.581) *large*	0.196 (0.102) *medium*	0.528 (0.025) *small*

**COD_def_**	0.80 ± 0.08	0.82 ± 0.08	2.87	0.79 ± 0.14	0.75 ± 0.09	-4.08	0.558 (0.022) *small*	0.427 (0.040) *small*	0.127 (0.139) *large*

Abbreviations: LSG: linear sprint group; CSG: curve sprint group; M505 COD Test: modified 5-0-5 change of direction test; COD_def_: change of direction deficit.

** Significant differences (p < 0.01) between pre-and post-test.

* Significant differences (p < 0.05) between pre-and post-test.

### LS performance

Regarding LS performance, the between-group analysis revealed no significant time × group interactions (*p* > 0.05) for all split times. In the within-group analysis, there were significant effects for time in the 10 m (F = 17.398, *p* < 0.001), 15 m (F = 21.021, *p* < 0.001), 20 m (F = 21.022, *p* < 0.001), and 30 m (F = 27.570, *p* < 0.001) sprint test. For the LSG, the results showed significant small to moderate enhancements in 10 m (*d* = -0.63), 15 m (*d* = -0.62), 20 m (*d* = -0.67), and 30 m (*d* = -0.59) sprint performance between the pre- and post-intervention. In reference to the CSG, the statistical analyses revealed significant small improvements in 10 m (*d* = -0.58), 20 m (*d* = -0.47), and 30 m (*d* = -0.52) sprint performance after the intervention period.

### CS performance

In reference to CS performance, the statistical analysis revealed significant time×group interactions in CS good (F = 10.222, *p* = 0.006) and weak side (F = 10.220, *p* = 0.006). Additionally, significant time effects were observed in the good side (F = 9.435, *p* = 0.007) and weak side (F = 8.760, *p* = 0.009) CS test. Specifically, the CSG exhibited moderate improvements in the CS good side (*d* = -1.05) and weak side (*d* = -0.90) performance.

### COD performance

In the modified 505 test, a significant main effect for time was observed (F = 22.182, *p* < 0.001). Regarding the LSG, there were small (*d* = -0.44) enhancements in COD performance between the pre- and post-test. In this regard, the CSG presented moderate (*d* = -0.93) improvements between the pre- and post-test. Nevertheless, no-significant time × group interaction was found in COD test performance (F = 0.417, *p* = 0.528).

### Horizontal FV profile

**FIG. 2:**
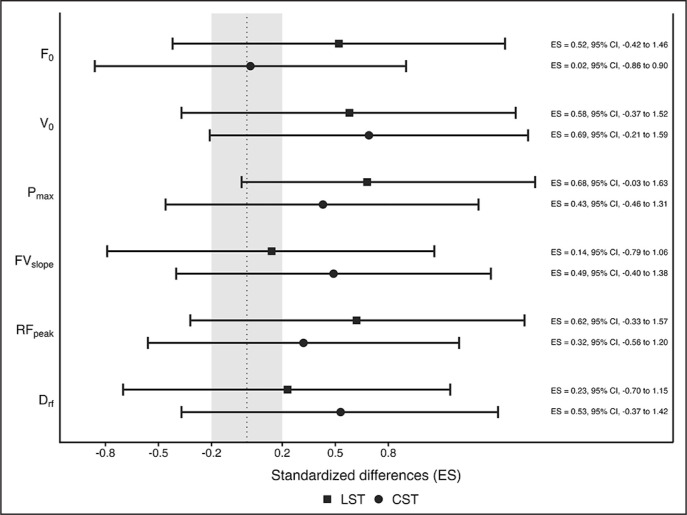
Standardized differences (95% CI) in horizontal F-V profile variables between pre-and post-test for linear (filled squared) and curvilinear (filled circles) sprint training groups. LST: linear sprint training group; CST: curvilinear sprint training group; ES: effect size; F_0_: maximal theoretical horizontal force; V_0_; maximal theoretical running velocity; P_max_: maximal power output; FV slope: slope of horizontal FV profile; RF_peak_: proportion of total force production in the forward direction; D_RF_: decrease in the ratio of force with increasing running speed.

Table 5 shows mean values, SD, and percentage of change, while Figure 2 presents standardized differences between pre- and post-intervention for horizontal F-V profile variables. No significant time × group interactions (p > 0.05) were observed for any mechanical sprinting variable. However, the within-group analysis revealed significant time effects for V_0_ (F = 9.987, *p* = 0.006), P_max_ (F = 27.776, *p* < 0.001), and RF_peak_ (F = 10.645, *p* = 0.005). The results indicated small to moderate improvements in the LSG from pre-to-post-intervention for V_0_ (d = 0.58), P_max_ (d = 0.68), and RF_peak_ (d = 0.62). In contrast, the CSG showed only small significant enhancements in P_max_ (*d* = 0.53) from the pre- to post-test.

**TABLE 5:** Changes in horizontal F-V profile after five weeks of sprint training in youth soccer players.

	LSG			CSG			ANOVA *P* values ($\eta_{\text{p}}^{2}$) ($\eta_{\text{p}}^{2}$ category)		

	Pre	Post	∆ (%)	Pre	Post	∆ (%)	Time	Group	Time × Group
**F_0_** (N/kg)	5.35 ± 0.38	5.56 ± 0.42	4.20	5.72 ± 0.60	5.73 ± 0.27	0.83	0.298 (0.064) *medium*	0.125 (0.133) *medium*	0.352 (0.051) *small*

**V_0_** (m/s)	9.25 ± 0.95	9.92 ± 1.34^**^	7.31	8.93 ± 0.52	9.40 ± 0.81	5.22	0.006 (0.370) *large*	0.300 (0.063) *medium*	0.586 (0.018) *small*

**P_max_** (W/kg)	12.42 ± 1.89	13.78 ± 2.13^**^	11.33	12.80 ± 1.68	13.50 ± 1.60^*^	5.80	< 0.001 (0.620) *large*	0.952 (0.001) *trivial*	0.118 (0.137) *medium*

**FV slope**	-0.58 ± 0.05	-0.57 ± 0.09	-1.53	-0.64 ± 0.07	-0.61 ± 0.05	-3.98	0.274 (0.070) *medium*	0.038 (0.230) *large*	0.576 (0.019) *small*

**RF_peak_** (%)	37.51 ± 2.37	38.93 ± 2.19^**^	3.93	38.59 ± 2.44	39.26 ± 1.68	1.87	0.005 (0.385) *large*	0.470 (0.031) *small*	0.254 (0.076) *medium*

**D_RF_** (%)	-5.48 ± 0.48	-5.32 ± 0.88	-2.63	-6.03 ± 0.63	-5.74 ± 0.46	-4.07	0.219 (0.087) *medium*	0.049 (0.208) *large*	0.693 (0.009) *trivial*

Abbreviations: LSG: linear sprint group; CSG: curve sprint group; F_0_: maximal theoretical horizontal force; V_0_: maximal theoretical running velocity; P_max_: maximal power output; FV slope: slope of horizontal FV profile; RF_peak_: proportion of total force production in the forward direction; D_RF_: decrease in the ratio of force with increasing running speed.

** Significant differences (p < 0.01) between pre-and post-test.

* Significant differences (p < 0.05) between pre-and post-test.

## DISCUSSION

This study analysed the effects of LST compared to CST with an equivalent session volume on LS and CS performance, horizontal F-V profile, and COD ability in young soccer players. To the author’s knowledge, this is the first study to explore the different adaptations induced by 6-week sprint training with different trajectories (linear vs. curve) on physical fitness. The main findings of this study indicate enhanced LS performance following both training conditions, with the LSG showing superior effects. Second, CS performance significantly improved only following the CSG. Third, both training conditions resulted in enhanced COD performance, with the CSG having slightly greater effects. Finally, the LSG had greater improvements in horizontal FV profile parameters than CSG. These results suggest that the application of both training regimes during sprint training programmes might play a crucial role in enhancing multidirectional speed performance in youth soccer players.

Previous research has indicated that sprint performance seems to develop due to anatomical growth and maturation processes, although sprint training is required in order to maximize speed development [33, 34]. In that vein, a recent meta-analysis conducted by Moran et al. [35] indicated that maturation could influence the dose-response effectiveness related to primary sprint training methods, reporting greater benefits for mid- and post-peak height velocity (PHV). Additionally, recent systematic reviews and a meta-analysis concluded that primary training methods, such as sprint technique training and unresisted sprint training, do not significantly enhance short and long-sprint performance in football code athletes [17, 18]. Conversely, our findings align with Rey et al.’s [28] research, showing significant improvements (range from -6.6% to -2.9%) in 10, 20, and 30 m sprint performance following six weeks of short versus long-distance unresisted LST in U19 male soccer players. Similarly, Marzouki et al. [29] found small to moderate increases (ES range from 0.48 to 0.77) in these distances after a 10-week training period in young male players participating at the junior regional level, regardless of the weekly frequency (1 vs. 2 sessions). Thus, the observed positive effects in these studies may be influenced by the player’s training status, as previous research has suggested that non-elite players may benefit more from training than elite players. For the first time, this study assessed the effect of the trajectory (LST vs. CST) during sprinting on the horizontal F-V profile. While the CSG only improved in P_max_, the LSG showed greater changes in V_0_, P_max_, and RF_peak_. These changes contrast with a pilot study [36] which concluded that linear unresisted sprint training did not enhance the horizontal FV profile in adult male soccer players. From a biomechanical standpoint, CS involves increased ground contact time, decreased mean race velocity, decreased resultant ground reaction forces, and different force orientations compared to LS [16, 37–39]. These CS characteristics may hinder players from displaying their maximal neuromuscular capacities, which have been a predictor of LS [40], potentially explaining the lack of a significant transfer effect on the horizontal FV profile parameters.

Time motion analysis in soccer matches revealed that most sprint efforts follow a curvilinear trajectory, regardless of player position [11, 41]. Thus, strength and conditioning coaches should design sprint-specific drills and training programmes based on CS demands. In that sense, this is the first study analysing the effects of sprint training on CS performance, so a direct comparison with previous scientific literature is not possible. Notably, only the CSG demonstrated moderate improvements in CS performance for both the good side (ES = 1.05) and weak side (ES = 0.90) in young soccer players. Considering the principle of specificity, it seems reasonable to suggest that sprint training protocols with curvilinear elements appear more effective in enhancing CS abilities than traditional LST. This notion is reinforced by McBurnie & Dos’Santos [42], who recommend assessing and training CS as a distinct athletic quality in young soccer players. Importantly, to ensure uniform lower-limb adaptations and account for the distinct roles of the inside and outside leg in CS, we maintained an equal total distance per session for both clockwise and anticlockwise CS [14] According to soccer competitive demands, multidirectional sprint programmes could include CST to help youth soccer players adjust technically and optimize force application [14, 42, 43]. However, due to limited scientific evidence, further research is necessary to clarify the impact of LST strategies on CS performance.

COD is a crucial attribute linked to success in offensive and defensive soccer actions [7, 44, 45], with the potential to distinguish skill levels and playing standards [46, 47, 48]. Recent systematic reviews and a meta-analysis suggest that sprint training is effective for improving COD performance [49, 50]. In this study, both LSG and CSG demonstrated 2.3% and 3.1% improvements in the modified 505 test, respectively. However, the lack of significant differences between sprint training groups suggests that sprint trajectory may not be a determinant factor in COD improvement among youth soccer players. Fílter et al. [14] and Kobal et al. [51] found moderate to large associations of CS time with Zig-Zag test performance in female and male soccer players. Likewise, the underpinning mechanisms of COD ability gains could be related to the optimization stretch-shortening cycle, change in sprint acceleration kinematic parameters (i.e., step length, stride frequency, contact time), and horizontal propulsive force production (i.e., P_max_, RF_peak_) [52, 53]. In line with our findings, McMorrow et al. [54] reported a significant increase in 180º COD performance by 3.3% and 3.7% among professional soccer players following 6 weeks of in-season sprint training, both resisted and unresisted. Likewise, in a youth soccer player age group, Rey et al. [28] observed a significant 4.7% to 5.5% improvement in T-Test performance after 6 weeks of unresisted sprint training, with varying distances. Due to the lack of studies assessing the effects of CS on COD performance, further studies are needed to corroborate these findings.

This study has limitations that must be acknowledged. Firstly, the small sample size (i.e., 19 young soccer players) could limit the power of this study, although it was similar to previous sprint training studies. Furthermore, the sample was composed of only one soccer academy, so the current results might not be generalizable for other soccer academy backgrounds, or competitive levels. Secondly, the training intervention only comprised 6 weeks, which is a relatively brief time period to optimize training adaptations. However, the duration of the sprint training protocol was enough to obtain enhancements in young soccer players. Thirdly, the study design did not include a control group, which could limit the conclusions. Furthermore, the study did not record data to quantify the intensity of training sessions (e.g., rating of perceived exertion scales). Finally, both sprint groups could have been affected by potential learning effects related to their force vector specific trajectory. To provide more conclusive results, future studies should attempt to use a larger sample size, longer-term interventions, a control group, and a rating of perceived exertion scale.

## CONCLUSIONS

This research was the first to investigate the effects of CST on LS and CS performance, and horizontal force-velocity profile. The findings indicated that both CST and LST elicited trajectory-specific adaptations. LST showed greater improvements in LS performance (10, 20, 30 m sprint time) and the horizontal F-V profile parameters, while CST resulted in greater COD performance. Importantly, only CST significantly enhanced CS performance for both sides. Therefore, our current findings suggest that based on soccer’s demands, strength and conditioning coaches should integrate both LST and CST methods to effectively prepare soccer players and enhance their sprinting performance.
